# Real-Time Assessment of Stress and Stress Response Using Digital Phenotyping: A Study Protocol

**DOI:** 10.3389/fdgth.2020.544418

**Published:** 2020-10-15

**Authors:** Stephan T. Egger, Marius Knorr, Julio Bobes, Abraham Bernstein, Erich Seifritz, Stefan Vetter

**Affiliations:** ^1^Department of Psychiatry, Psychotherapy and Psychosomatics, Faculty of Medicine, Psychiatric University Hospital of Zurich, University of Zurich, Zurich, Switzerland; ^2^Department of Psychiatry, Faculty of Medicine, University of Oviedo, CIBERSAM, Oviedo, Spain; ^3^Department of Informatics, University of Zurich, Zurich, Switzerland

**Keywords:** stress, cortisol, trier social stress test, digital phenotyping, ecological moment assessment

## Abstract

**Background:** Stress is a complex phenomenon that may have a negative influence on health and well-being; consequently, it plays a pivotal role in mental health. Although the incidence of mental disorders has been continuously rising, development of prevention and treatment methods has been rather slow. Through the ubiquitous presence of smartphones and wearable devices, people can monitor stress parameters in everyday life. However, the reliability and validity of such monitoring are still unsatisfactory.

**Methods:** The aim of this trial is to find a relationship between psychological stress and saliva cortisol levels on the one hand and physiological parameters measured by smartphones in combination with a commercially available wearable device on the other. Participants include cohorts of individuals with and without a psychiatric disorder. The study is conducted in two settings: one naturalistic and one a controlled laboratory environment, combining ecological momentary assessment (EMA) and digital phenotyping (DP). EMA is used for the assessment of challenging and stressful situations coincidentally happening during a whole observation week. DP is used during a controlled stress situation with the Trier Social Stress Test (TSST) as a standardized psychobiological paradigm. Initially, participants undergo a complete psychological screening and profiling using a standardized psychometric test battery. EMA uses a smartphone application, and the participants keep a diary about their daily routine, activities, well-being, sleep, and difficult and stressful situations they may encounter. DP is conducted through wearable devices able to continuously monitor physiological parameters (i.e., heart rate, heart rate variability, skin conductivity, temperature, movement and acceleration). Additionally, saliva cortisol samples are repeatedly taken. The TSST is conducted with continuous measurement of the same parameters measured during the EMA.

**Discussion:** We aim to identify valid and reliable digital biomarkers for stress and stress reactions. Furthermore, we expect to find a way of early detection of psychological stress in order to evolve new opportunities for interventions reducing stress. That may allow us to find new ways of treating and preventing mental disorders.

**Trial Registration:** The competing ethics committee of the Canton of Zurich, Switzerland, approved the study protocol V05.1 May 28, 2019 [BASEC: 2019-00814]; the trial was registered at ClinicalTrials.gov [NCT04100213] on September 19, 2019.

## Background

Stress is a complex natural phenomenon, broadly defined as “the non-specific response of the body to any demand” (1). Oversimplified, this response can be divided into two components: the physiological reaction on the one hand and the subjective experience on the other (2, 3). Physiological stress causes the liberation of hormones (mainly adrenalin and cortisol) and the activation of the autonomic nervous system (1–6), resulting in changes in several physiological variables, including heart rate, heart rate variability, respiratory rate, skin conductance, and temperature (3–5, 7). The response on the behavioral level varies greatly; broadly, it may be conceived as a freeze, flight, fight, fright, or faint response (8).

So far, many studies demonstrate the negative influence of psychological stress on health and well-being (7) with several somatic and even some psychiatric disorders etiologically linked to stress (6, 9). Furthermore, mental disorders are generally conceived as harmful dysfunctions of psychological coping and adaptation mechanisms (10). For nearly three decades, the incidence of mental disorders has been continuously rising worldwide (11, 12), and this consistently accounts for a substantial proportion of social costs and the burden of disease (11, 12). The increment of psychiatric disorders has been attributed in Western societies to the rise in stress levels. The development of methods to either prevent psychiatric disorders or significantly improve their outcome has, by contrast, been slow (12).

Digital technology and information sciences are expected to profoundly change the way we understand and approach mental health (13), for example, the ubiquitous presence of smartphones (13) and the increasing availability and affordability of wearable devices capable of measuring bodily functions (14). Digital phenotyping (DP) seeks to find digital biomarkers, particularly for cognition, stress, and behavior (13, 15–19), by assessing smartphone interaction and voice and speech features, together with monitoring movement and physiological parameters (20, 21). However, from current studies (15, 17), together with earlier psychological studies (22, 23), it becomes clear that a proper validation of the users' individual emotional experience is essential (13, 16, 24–26).

Through the DP of physiological and psychological stress reactions, in real-life situations and a controlled laboratory setting, in a population of healthy participants and patients with a psychiatric disorder, we expect to find reliable and valid digital biomarkers. Therefore, we plan to conduct a psychological and physiological study, combing ecological momentary assessment (EMA) and a laboratory psychological paradigm to induce stress, namely the Trier Social Stress Test (TSST) (27).

## Methods/Design

The aim of the present trial is to establish a relationship between the physiological parameters measured by commercially available wearable devices and changes in cortisol levels obtained during everyday difficult and stressful situations and a controlled stress situation. We expect to establish a valid and reliable DP for stress and stress reactions as well as for patients with a psychiatric disorder and otherwise healthy subjects.

### Participants

**Figure 1:**
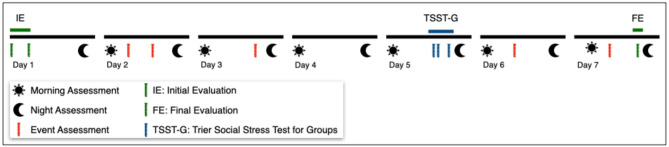
Study Outline. The initial and final evaluations (green) include a psychological test battery and the collection of cortisol samples. Well-being and basal cortisol levels are assessed daily at fixed time frames (only morning and night assessment shown). During the TSST-G (blue) DP, stress and cortisol levels are conducted. Coincidentally experienced challenging of stressful situations/events (red) are assessed shortly after they occur (shown for illustrative purposes only). Physiological parameters are continuously assessed through wearable devices.

**Figure 2:**
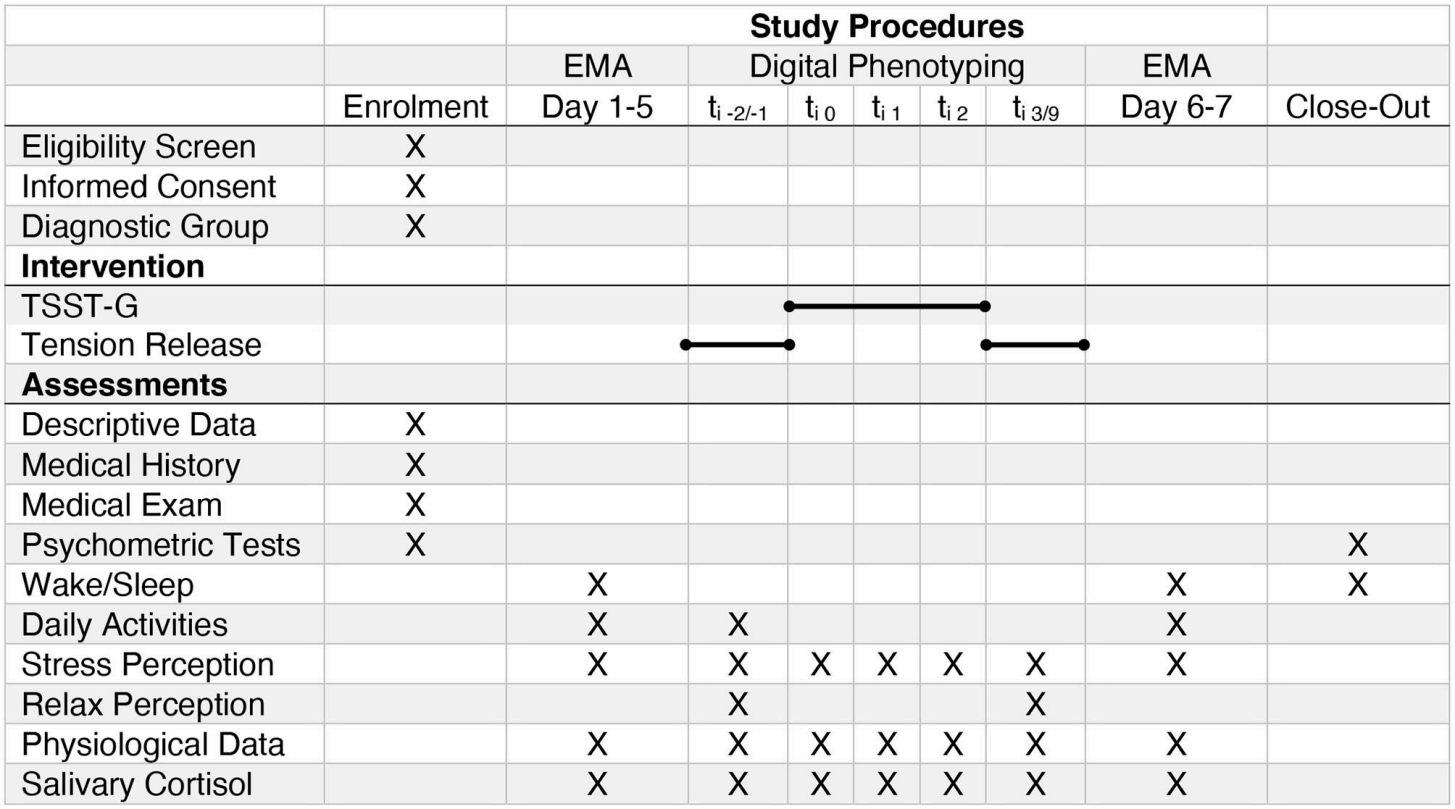
SPIRIT Study Schedule (EMA, Ecological Momentary Assessment; TSST-G, Trier Social Stress Test for Groups). The bar denotes the different parts of the intervention (compare Figure 3). t_i−2/−1_ briefing and tension release exercise previous to the TSST-G; t_i1_TSST-G Speech; t_i2_TSST-G Math; t_i3/9_TSST-G debriefing and tension release.

Participants include cohorts of participants with and without a psychiatric disorder; those with a psychiatric disorder are further categorized according to diagnosis into internalizing, externalizing, or psychotic (thought) disorders. To ensure generalizability of the findings and to minimize confounders, an overall physically healthy sample is crucial. Another critical factor is hand preference because it can influence the measurement and, therefore, reduce generalizability (28); for convenience, we include only right-handed persons. The inclusion and exclusion criteria are summarized in Table 1, and they are determined through the collection of a complete medical (and psychiatric) history and a medical exam (Figures 1, 2). All participants undergo the same procedures, regarding psychometric screening and profiling, EMA, DP, and the TSST for groups.

**Table 1:** Inclusion and exclusion criteria for trial participation.

**Inclusion criteria**
Participants are between 18 and 65 years of age
Participants are competent to give informed consent
Right-handedness as determined by the Hand Preference Questionnaire (HPQ) (29)
German language proficiency as a native speaker or level B1 according to the Common European Framework of Reference for Languages (CEFRL) (30)
Diagnosis of a cluster C personality disorder according to ICD-10 (31); or
Diagnosis of a depressive disorder according to ICD-10 (31); or
Diagnosis of schizophrenia or schizoaffective disorder according to ICD-10 (31); or
Without a current psychiatric disorder
**Exclusion criteria**
Low Intelligence as confirmed by failure to complete regular compulsory education
Pregnancy or over 2 weeks delay in the menstrual cycle
Previous participation in a psychological trial involving psychosocial stress assessment
Current neurological disorder
Current cardiovascular disorder
Current respiratory disorder
Current substance use or withdrawal
Any change in medication in the previous week

### Psychometric Measurements

All participants (regarding their psychiatric condition) undergo a full psychological screening and profiling with a standardized psychometric test battery, including self-administered and observational instruments. Raters of the following are psychiatry residents or clinical psychologists. They are trained in specific workshops on the use and objectives of the measures used in the study. The workshops follow a standardized schedule, using case vignettes and video examples. Refresher training sessions are provided regularly with trainers available for consultation at any time. The psychometric instruments included in the test battery are summarized in Table 2.

**Table 2:** Psychometric measurement instruments included in the Test Battery used in the study.

**Instrument**	**Description**
Brief Neurocognitive Assessment (*BNA*)	The Brief Neurocognitive Assessment (*BNA*) was developed as a brief, easily applicable and reliable tool to evaluate global neurocognition and impairment, primarily in patients with a diagnosis of schizophrenia (32)
Brief Symptom Inventory (*BSI*)	The Brief Symptom Inventory (*BSI*) (33) is a self-administered questionnaire assessing psychological distress; it can be used either for screening or outcome evaluation
Clinical Global Impression *(CGI)*	The Clinical Global Impression *(CGI)* scale was initially introduced in psychopharmacological trials (34). It is a brief, easy-to-use, and pragmatic tool for the assessment of psychiatric illness severity and changes over time (35)
Global Assessment of Functioning (*GAF*)	The Global Assessment of Functioning (*GAF*) is widely used in psychiatric research. It is a single-item observer-rated scale of overall functioning on a continuum from mental health to mental illness (36)
Hamilton Anxiety Rating *(HAM-A)*	The Hamilton Anxiety Rating Scale *(HAM-A)* comprises 14 items and provides an overall measure of anxiety, including psychological, cognitive, and somatic symptoms (35, 37). The scale can be used to measure anxiety in various psychiatric conditions (35)
Hamilton Depression Scale *(HAM-D)*	The Hamilton Depression Rating Scale *(HAM-D)* is a checklist of 21 items designed to measure the severity of depression (35, 38). Besides depression, it has also been used to measure depressive symptoms in other disorders (35)
Health of the Nation Outcome Scales (*HoNOS*)	The Health of the Nation Outcome Scales (*HoNOS*) is an observer-rated scale to assess the severity of a psychiatric disorder in four dimensions: behavior, impairment, symptoms, and social problems (39, 40)
Insecurity Questionnaire *(IQ-24)*	The Insecurity Questionnaire [German: Unsicherheitsfragebogen] *(IQ-24)* is a self-administered questionnaire with 24 items developed to assess insecurity (41)
Mini ICF- APP *(mICF)*	The Mini ICF-APP *(mICF) is* a short observer-rated scale to assess the level of functioning and capacity. It is easy to use and possesses good psychometric properties (42, 43)
Mini-International Neuropsychiatric Interview *(MINI)*	The Mini-International Neuropsychiatric Interview (MINI) (44) is a structured diagnostic interview. It was designed as a quick but accurate structured psychiatric diagnostic interview (according to DSM-IV and ICD-10) for clinical trials and epidemiology studies
Montreal Cognitive Assessment (*MoCA*)	The Montreal Cognitive Assessment (MoCA) (45, 46) is a brief cognitive screening tool to detect mild cognitive impairment in patients performing in the normal range on the Mini-Mental Status Exam
Positive and Negative Syndrome Scale (*PANSS*)	The Positive and Negative Syndrome Scale (*PANSS*) is a semistructured interview designed to measure the severity of psychopathology in patients with a psychotic disorder: mainly schizophrenia and schizoaffective disorder (35, 47). The PANSS measures symptoms in three domains: positive, negative, and general symptoms
Protocol for Sleep Examination *(PSE)*	The Protocol for Sleep Examination [German: Abend/Morgenprotokolle für die Schlafuntersuchung] *(PSE)* was developed to assess the subjective dimension of sleep, daily activities, and distress. It is divided into morning and night (bedtime) sections (48)
Short Stress Questionnaire *(SSQ)*	The Short Stress Questionnaire [German: Kurzfragebogen zur aktuellen Beanspruchung] *(SSQ)* was developed to assess subjective levels of tension or stress associated with a current task, situation, or experience (49)
Toronto Alexithymia Questionnaire *(TAQ)*	The Toronto Alexithymia Questionnaire *(TAQ)* (50, 51) was developed to assess difficulties identifying subjective emotional feelings, distinguishing between feelings and the bodily sensations of emotional arousal and difficulty describing feelings to other people (51, 52)
Yale-Brown Obsessive-Compulsive Scale *(Y-BOCS)*	The Yale-Brown Obsessive Compulsive Scale *(Y-BOCS)* was developed to measure the severity of obsessive-compulsive symptoms; these are rated in terms of time spent on such activities, interference with functioning, distress, resistance, and control (35, 53)
Young Mania Rating Scale *(YMRS)*	The Young Mania Rating Scale *(YMRS)* is an observer-rated checklist, measuring manic symptoms to quantify the severity and the effect of treatment (35, 54). It includes the core symptoms of mania occurring in both mild and severe illness.

### EMA and DP

The phenomenological assessment usually relies on a first-person narrative account collected at research or clinical visits. Self-reports, however, are known to sometimes be inaccurate for several reasons, for example, that events fade from memory over time. In contrast, EMA allows the timely record of a person's experience and behavior in the natural environment, thus, increasing the validity and allowing the inference of factors influencing behavior and experience. EMA is a long-known methodology in psychological and anthropological research, usually with the use of dairies or logbooks. The appearance of smartphones and wearable devices facilitates the implementation of EMA studies (55, 56).

EMA is conducted over a whole week using a custom smartphone application and two wearable devices. Through the smartphone application, participants are able to evaluate their daily activities and sleep. In addition, the application prompts the participants once or twice a day about their current activity. Participants are able to log any stressful and challenging situation. Participants have to answer a short questionnaire regarding their current activity, well-being, and stress level (see Figures 1, 2). Through two commercially wearable devices (Vívosmart® wristband and Everion® armband), several physiological parameters are continuously monitored and recorded, including heart rate, skin conductance, temperature, movement, and acceleration (see Table 3). We included two devices in order to allow for comparison and generalizability of the results, especially taking into account possible flaws in the use and the measurement quality of the devices (57).

**Table 3:** Digital parameters for Ecological Momentary Assessment (EMA) and Digital Phenotyping (DP).

**Parameter**	**Smartphone**	**Everion^®^**	**Vívosmart^®^**
Heart rate		X	X
Heart rate variability		X	X
Blood pulse wave		X	
Respiratory rate		X	
Oxygenation		X	
Skin blood perfusion		X	
Skin temperature		X	
Electrodermal activity		X	
Activity/movement	X	X	X
Distance	X		
Altitude	X		X
Location	X		

Cortisol secretion follows a circadian rhythm, usually with a peak in the morning and slowly declining throughout the day with variations from day to day and individual to individual (58). Therefore, for proper validation and interpretation, regular measurements of cortisol levels are necessary (59, 60). Participants collect a saliva sample four times a day (morning, midday, afternoon, and night); after experiencing a difficult or stressful situation and at random once or twice a day. Saliva samples are picked up and sent once a day (at night) to the laboratory for the quantification of cortisol levels, and after analysis, samples are destroyed.

### Trier Social Stress Test for Groups (TSST-G)

**Figure 3:**
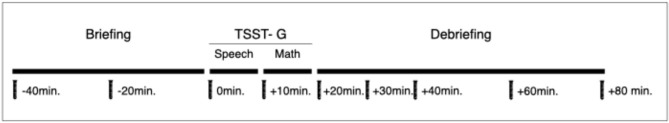
Outline for the Trier Social Stress Test (for Groups). Physiological parameters are continuously monitored. At each time point, a psychological stress response assessment takes place and a saliva cortisol sample is collected.

The TSST is an extensively used and well-validated psychological paradigm to induce psychobiological stress in laboratory settings (61–64) with a significant association with an acute stress response in real life (62, 65, 66). The TSST has been modified in order to be conducted in groups; in our current study, we include three to five participants (from the same diagnostic group), using TSTT-G procedures analogous to previous studies (67–69). The TSST-G consists of three phases: a briefing, the psychological test itself, and a debriefing. The phases last 40, 20, and 60 min, respectively (Figures 2, 3). An experienced psychotherapist conducts the briefing and debriefing of the TSST-G. The TSST-G itself is conducted by personnel unknown to the participants. During the TSST-G, saliva samples are obtained at regular intervals and cortisol levels measured.

Each participant undergoes an individual briefing phase. Participants are required to prepare a speech for a job application. After a few minutes, participants are accompanied into the test room and are seated next to each other, separated by partitions in order to avoid eye contact. They are told that an expert committee will conduct an analysis of their performance and that they will be videorecorded (no actual recording is performed) for further analysis. The participants present their speech (2–3 min each) in a previously set random order. Next, the participants conduct a subtraction task (for 2 min) as quickly and as accurately as possible. If participants make a mistake, they are asked to start over again. The order of participation once again is random. Once the last participant has completed the task, the committee leaves the room. Participants are accompanied back to the preparation room, where they are debriefed and may engage in any relaxing activity for 60 min.

### Sample Size Calculation and Statistical Analysis

Previous research has consistently shown that the TSST significantly increases the cortisol levels with moderate effect sizes regarding baseline (63). Therefore, we expect a low to moderate effect size in cortisol through the TSST-G in our study. We calculate our required sample size using G^*^Power 3.1 (70) (ANOVA: repeated measures, within and between factors; effect size *f* = 0.4; α = 0.05; power = 0.8; number of groups = 4, number of measures = 9, nonsphericity correction = 0.125). Based on that calculation, at least 24 participants per group are required to detect moderate-sized differences: to improve capacity, we include at least 30 participants in each group. Only data sets of participants who complete the intervention are considered (completed TSST-G and at least 70% completion of the EMA); therefore, recruitment continues until the number of participants for each group is reached. Already enrolled participants are able to complete the study.

The primary analysis is conducted with complete cases only; dropouts are replaced by recruiting new subjects. Secondary analysis includes incomplete cases and dropouts. If a participant withdraws from the study, his or her data is anonymized and his or her name is deleted permanently from all study records. Unless otherwise stated, his or her remaining data is used in the secondary analysis. Data analysis does not pursue hypothesis testing; through the statistic scrutiny of the data, we aspire to gain a better understanding of the possibilities offered by wearable devices for the assessment of stress and stress reactions and finding digital biomarkers. Accordingly, the findings of the study serve for the formulation of hypothesis and hypothesis testing in future studies.

The demographic and clinical characteristics of the sample are compared at baseline using an ANOVA, excluding gender, which is analyzed using the chi-square test. Repeated-measures multivariate analyses of variance (MANOVAs) are used to assess changes in symptomatology, functionality, cognition, and physiological parameters. To infer differences in stress reactions according to the subjective experience and clinical characteristics, we use a multivariate regression analysis as well as time series analysis. To avoid inflation of type II errors, we apply a Bonferroni correction for multiple comparisons. The significance threshold is set at 0.05. Cohen's *d* is calculated to determine the effect size (71). Multiple and logistic analyses as well as time series analysis is performed. Due to the complexity of the data, with a large number of variables and potential confounders, a machine learning algorithm is used to detect complex relationships between the stress, psychopathology, and physiological measures (72).

For each wearable device, machine learning is conducted stepwise, using a supervised learning approach at first and a deep learning approach at last. For analysis, three separate data sets are created. The first data set comprises the measures collected during the TSST-G with the speech and math as stress events and the briefing and debriefing as relaxing events. This data set is subdivided into two sets: one for training the model and one for testing. One stress and one relaxing event are randomly assigned to either one of the data sets. The second data set consists of the three full-day measurements selected at random: two from the days previous and one from the days after the TSST-G. The second data set is used for the deep learning algorithm for the detection of stress and relaxation. The third and final data set comprises the remaining days: two previous and one after the TSST-G. This data set will be used for testing the obtained models.

### Quality of Data, Missing Data

The design of our trial, with the preparation of the probands and instruments, allows us to ensure that measurements obtained during the TSST-G have high quality with a low artifact rate. Due to the complexity and duration of the remaining intervention, we cannot rule out that all the measurements obtained will reach a high-quality threshold. The use of a device (Everion®) with a high measurement quality as well as its placement (57) should increase the quality of the measurements. Missing measurements and artifacts from the digital devices are not replaced. Missing items in the different psychometric instruments are replaced according to the rules and conventions for each instrument.

## Trial Status

The competing ethics committee of the Canton of Zurich, Switzerland, approved the study protocol V05.1- May 28, 2019 [BASEC: 2019-00814]; the trial was registered at ClinicalTrials.gov [NCT04100213] on September 19, 2019. Recruitment starts in Fall/Winter 2020. We expect to recruit the whole sample in 9 to 12 months from the first enrollment.

## Discussion

Stress is a known risk factor for several, if not all, psychiatric disorders. However, the perception and reaction to stress show considerable variability among the general population and even more among those suffering from a psychiatric disorder. Healthy subjects are more or less consciously aware of stress and potentially stressful situations and, therefore, able to adjust and modify their behaviors in order to master life's challenges. Patients with a psychiatric disorder, conversely, have a disrupted perception, awareness, and reaction to stress (2, 73, 74), hampering them in adapting and coping with everyday demands. Stress has, therefore, become a major target of lifestyle and well-being and psychiatric prevention and treatment research with several interventions focusing on stress awareness and management.

The use of smartphones and wearable devices nowadays is ubiquitous with a significant increase in their application to monitor psychological well-being and stress. Uncountable digital services are claiming to appraise and improve physical and psychological well-being (26, 75). However, despite gaining popularity, their use remains controversial. Users frequently experience deception (76, 77), generally due to privacy and confidentiality issues (25, 76, 78, 79) but also inaccurate feedback or even dangerous advice (25, 26, 79).

There is still a lack of guidance in the use of such devices in general and in psychiatry in particular with guidelines and legal regulations that are still emerging (61, 80). From the available services, only a tiny fraction has been validated adequately in controlled studies (24, 26, 81). Persons with a psychiatric disorder are under-represented in current studies, reducing the use and applicability of such devices in psychiatric settings. Their reckless use may be detrimental, dangerous, or even harmful (81, 82).

From current digital trials (15, 17) and earlier psychological studies (22, 23), it is clear that proper validation and fitting to the users' individual emotional experience is required (13, 16, 24–26). We consider it essential to assess the individual stress and stress reactions in everyday situations and a controlled laboratory setting. The TSST (27) is an extensively used and well-validated psychological paradigm to induce psychobiological stress (61–64) with a significant association with acute stress response in real life (62, 65, 66).

In order to establish valid and reliable digital biomarkers, the study population is crucial (16, 20). Psychiatric diagnoses have overlapping symptoms and high psychiatric comorbidity (74, 83), making it challenging to form homogeneous groups. Therefore, in our study, we aim to establish a complete psychological profile (beyond individual diagnoses) of the participants with a transdiagnostic test battery, including the assessment of threshold and subthreshold psychiatric symptoms. Likewise, we assess their psychosocial functioning, well-being, level of stress, and coping with the challenges of daily life. Cortisol release shows variations between and within individuals (58).

The regular sampling of cortisol in saliva during a whole week allows us to establish the cortisol secretion profile for each participant; the TSST-G allows us to establish the cortisol release during a standardized, controlled, and validated psychobiological stress paradigm, therefore, giving us a “fisheye perspective” on stress and stress reaction. We expect that day-to-day situations experienced as challenging and stressful enact a similar cortisol release and physiological response as the TSST-G. We anticipate that well-being and certain psychopathological states modify the individual's self-awareness and, therefore, the perception and reaction of challenging and stressful situations. The combination of psychopathological profiling, assessment of the subjective stress experience, physiological monitoring, and psychological observation during everyday life and under controlled and standardized laboratory conditions, however, provides a panoramic view, which, in turn, allows us to determine reliable and valid digital biomarkers.

The digital biomarkers we expect to find have the potential to facilitate self-monitoring of stress as well to serve as part of our diagnostic and therapeutic instruments. The use of both devices (high-quality and over-the-counter) allows inferring the suitability of this approach for daily use. The results obtained from this study serve for further hypothesis formulation and testing. Taking into account the complexity and dynamics in the field of digital technologies, the next step for testing and validating our results should take place in the frame of a citizen science project (84), simultaneously allowing the dissemination and improvement of the results of this study.

## Data Availability Statement

All datasets generated for this study are included in the article/supplementary material.

## Ethics Statement

The studies involving human participants were reviewed and approved by Ethics Committee of the Canton of Zurich [BASEC: 2019-00814]. The patients/participants provided their written informed consent to participate in this study.

## Author Contributions

SE: trial design, writing of the study protocol, and writing of the manuscript. MK: writing of the manuscript. JB and AB: trial design, writing of the study protocol, and correction of the manuscript. ES and SV: trial design, writing and registration of the study protocol, and correction of the manuscript. All authors contributed to the article and approved the submitted version.

## Conflict of Interest

The authors declare that the research was conducted in the absence of any commercial or financial relationships that could be construed as a potential conflict of interest.

## Abbreviations

ANOVA: Analysis of Variance
BNA: Neurocognitive Assessment
BSI: Brief Symptom Inventory
CEFRL: Common European Framework of Reference for languages
CGI: Clinical Global Impression Scale
EMA: Ecological Momentary Assessment
GAF: Global Assessment of Functioning
HAM-A: Hamilton Anxiety Rating Scale
HAM-D: Hamilton Depression Rating Scale
HoNOS: Health of the Nation Outcome Scales
HPQ: Hand Preference Questionnaire
IQ-24: Insecurity Questionnaire
MANOVA: Multivariate Analyses of Variance
mICF: mini ICF- APP
MINI: Mini International Neuropsychiatric Interview
MoCa: Montreal Cognitive Assessment
PANSS: Positive and Negative Syndrome Scale
PSE: Protocol for Sleep Examination
SSQ: Short Stress Questionnaire
TAQ: Toronto Alexithymia Questionnaire
TSST (-G): Trier Social Stress Test (for Groups)
Y-BOCS: Yale-Brown Obsessive Compulsive Scale
YMRS: Young Mania Rating Scale.
